# Chlamydial Type III Secretion System Needle Protein Induces Protective Immunity against* Chlamydia muridarum* Intravaginal Infection

**DOI:** 10.1155/2017/3865802

**Published:** 2017-03-26

**Authors:** Ekaterina A. Koroleva, Natalie V. Kobets, Dmitrii N. Shcherbinin, Naylia A. Zigangirova, Maxim M. Shmarov, Amir I. Tukhvatulin, Denis Y. Logunov, Boris S. Naroditsky, Alexander L. Gintsburg

**Affiliations:** Gamaleya Institute of Epidemiology and Microbiology, Ministry of Health of Russian Federation, Gamaleya Street 18, Moscow 123098, Russia

## Abstract

*Chlamydia trachomatis* imposes serious health problems and causes infertility. Because of asymptomatic onset, it often escapes antibiotic treatment. Therefore, vaccines offer a better option for the prevention of unwanted inflammatory sequelae. The existence of serologically distinct serovars of* C. trachomatis* suggests that a vaccine will need to provide protection against multiple serovars.* Chlamydia *spp. use a highly conserved type III secretion system (T3SS) composed of structural and effector proteins which is an essential virulence factor. In this study, we expressed the T3SS needle protein of* Chlamydia muridarum,* TC_0037, an ortholog of* C. trachomatis* CdsF, in a replication-defective adenoviral vector (AdTC_0037) and evaluated its protective efficacy in an intravaginal* Chlamydia muridarum* model. For better immune responses, we employed a heterologous prime-boost immunization protocol in which mice were intranasally primed with AdTC_0037 and subcutaneously boosted with recombinant TC_0037 and Toll-like receptor 4 agonist monophosphoryl lipid A mixed in a squalene nanoscale emulsion. We found that immunization with TC_0037 antigen induced specific humoral and T cell responses, decreased* Chlamydia* loads in the genital tract, and abrogated pathology of upper genital organs. Together, our results suggest that TC_0037, a highly conserved chlamydial T3SS protein, is a good candidate for inclusion in a* Chlamydia* vaccine.

## 1. Introduction

*Chlamydia trachomatis* is the most common sexually transmitted bacterial pathogen. It imposes serious health problems in humans and can cause severe complications such as pelvic inflammatory disease and ectopic pregnancy and infertility in women [1]. Although antibiotic therapy effectively eliminates acute infection, it does not always moderate an established pathology, and frequent asymptomatic courses of infection preclude early diagnosis and treatment. To overcome these problems, the development of a vaccine is highly desirable.* Chlamydia muridarum* has been extensively used to study the mechanisms of* C. trachomatis* pathogenesis and immunity in a mouse model [2]. Intravaginal inoculation of mice with* C. muridarum* can lead to infection in the lower and upper genital tract, which closely mimics the pathology induced by* C. trachomatis* in humans [3]. Both animal and human studies have established a vital role for T cell-mediated immunity, predominantly that of IFN-*γ*-producing CD4^+^ T cells, and the complementary role of humoral immunity in host resistance to chlamydial infection [4, 5]. CD8^+^ T cells have been shown to protect against infection when cultured* ex vivo* and transferred to naive animals, and immunization with recombinant vaccinia viruses expressing CD8^+^ T cell antigens from* C. trachomatis* conferred protection in mice [6]. Chlamydia vaccine research has led to the discovery of a large number of protective antigens [7–9]. Major outer membrane protein (MOMP) and, more recently, polymorphic membrane proteins (Pmps), both of which elicit antibody and cell-mediated protective immune responses, are widely studied as potent vaccine candidates. However, the antigenic variation in MOMP and Pmps suggests that evaluation of conserved proteins as vaccine candidates could be valuable, as this strategy has been successful for developing vaccines against other pathogens [10].

The type three secretion system (T3SS) is the predominant virulence factor in* Chlamydia*. It is required for cell invasion and is active at all life stages [11, 12]. Some T3SS proteins are surface-exposed and can be targeted by neutralizing antibodies. The T cell response to T3SS antigens was recently shown to be associated with protection against* C. trachomatis* infection in highly exposed women [13], and T3SS components have recently attracted attention as vaccine candidates against other pathogenic bacteria [14–17]. The* C. trachomatis *T3SS filament protein, CdsF, and its orthologs in other bacteria form the needles of injectisomes and are believed to facilitate the insertion of translocators into the host cell membrane [11, 12, 18]. CdsF is highly conserved, showing 95% sequence identity in the genus* Chlamydia.* It is abundant on bacterial surfaces, raising the possibility that a CdsF-based vaccine may induce a wide range of protection against all medically significant strains.

The ability of human adenoviruses to induce strong innate and adaptive immune responses makes them a powerful delivery system to induce an immune response against an encoded antigen. Adenovirus has a natural tropism for the mucosal epithelium, which makes it an ideal vector for vaccination against infections acquired* via* mucosal surfaces [19]. Intranasal immunization in different experimental settings has been reported to provide potent protection against intravaginal challenge [18, 20–22]. Replication-defective adenovirus vectors (rAds) are widely used for transgene expression in different tissues and gene-based immunizations. Effective vaccines against tuberculosis, malaria, influenza, and other important infectious diseases have been designed from rAds. The majority of rAd-based medical preparations are currently in clinical trials [23–27]. There is a large body of evidence indicating that rAd-derived vaccines are safe and efficacious [28–31]. Combination of adenoviral-vectored and protein-in-adjuvant vaccines in “prime-boost” regimens with the aim of enhancing both cellular and humoral immune responses has shown promising results in HIV and liver-stage malaria vaccine preclinical research [32–38]. Toll-like receptor (TLR) agonists are usually codelivered with antigens as adjuvants to enhance the immunogenicity of vaccine components [39].

In this study, we expressed the T3SS needle protein of* C. muridarum,* TC_0037, a CdsF ortholog, in a replication-defective adenoviral vector (AdTC_0037) and evaluated its protective efficacy in an intravaginal* Chlamydia* infection mouse model. To study the protective immunity of our vaccine candidate, we utilized a prime-boost immunization protocol with AdTC_0037 intranasal priming and subcutaneous boosting with recombinant TC_0037 and TLR4 agonist monophosphoryl lipid A (MPLA), mixed in a squalene nanoscale emulsion. We found that immunization with TC_0037 antigen induced specific humoral and T cell responses, decreased* Chlamydia* loads in both the lower and upper genital tract, and reduced the pathology of upper genital organs.

## 2. Materials and Methods

### 2.1. Bioinformatic Antigen Design

The nucleotide sequence of the* C. muridarum* TC_0037 gene was obtained from UniProtKB (Q9PLQ8). We performed an* in silico* analysis of the TC_0037 gene on the presence of a bacterial signal sequence (http://www.cbs.dtu.dk/services/SignalP/) and no bacterial signal sequence was found. We further optimized TC_0037 gene sequences for expression in mouse cells by modifying its codons with the two most frequent amino acid triplets. Frequent* Mus musculus* codons were defined using the http://www.kazusa.or.jp/codon/ database. As TC_0037 is abundant in bacterial cells and small in size (82 amino acids, 9 kDa), the N-terminal portion of this protein was bound to a 128-amino acid-sized N-terminal portion of mouse mannose-binding lectin (MBL) to obtain TC_0037 hexamers crosslinked with a collagen-like domain. The nucleotide sequence of mouse MBL was obtained from UniProtKB (P41317). The MBL-TC_0037 gene was synthesized by Evrogen (Moscow, Russia) in plasmid pAL-TA-MBL-TC_0037.

### 2.2. Generation of Recombinant Adenoviruses

**Figure 1:**
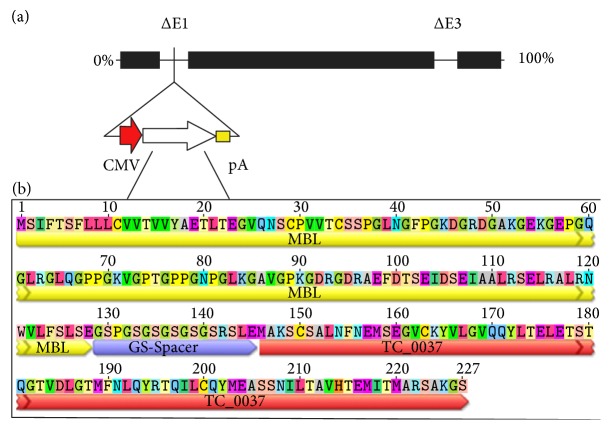
Design of* Chlamydia muridarum* TC_0037 expressed in an adenovirus construct. (a) Human recombinant adenovirus serotype 5 genome. CMV, cytomegalovirus promoter; pA, polyadenylation signal. (b) The amino acid sequence of chimeric antigen MBL-TC_0037.

The* Not*I and* Hind*III sites of the MBL-TC_0037 fragment were cloned into shuttle vector, pShuttle-CMV (cytomegalovirus), to obtain the shuttle plasmid, pShuttle-CMV-MBL-TC_0037. Homologous recombination was used to generate the replication-defective adenovirus, Ad-MBL-TC_0037 (Figure 1). For this purpose, the plasmid pShuttle-CMV-MBL-TC_0037 was linearized by* Pme*I, mixed with pAd-Easy (Adenoviral Vector System, StratoGen, New York, NY), and then cotransformed into* Escherichia coli* (BJ5183 strain). The obtained recombinant clones were used to extract plasmid DNA, whose molecular weight was later assessed.* E. coli* strain DH5alpha was transformed with plasmids larger than 20 kbp because this strain, unlike BJ5183, allows one to produce a sufficient amount of recombinant plasmids. The purified plasmid clones were analyzed by both cleavage with the* Hind*III restriction endonuclease and polymerase chain reaction (PCR). Next, we studied the infectivity of the described plasmids in permissive cells. HEK-293 cells were transfected with plasmid pAd-MBL-TC_0037 and linearized by* Pac*I. Transfection was performed in a 24-well plate using Lipofectamine 2000 (Invitrogen, Burlington, Canada). Ten days after the transfection, the cells were collected and subjected to a freeze-thaw cycle; the obtained lysate containing recombinant adenoviruses was used to infect HEK-293 cells in a 35 mm dish. After 5 days, specific lysis caused by cytopathic effect of the recombinant viruses was detected. The lysate was used to extract DNA and perform a PCR analysis. The cell lysate was shown to contain DNA of recombinant human adenovirus serotype 5 (rAd5) carrying insertions that encode the protective antigen.

### 2.3. Virus Accumulation

Recombinant adenovirus serotype 5 (rAd5) was grown in HEK-293 cells as described elsewhere [39]. Cell monolayers at 50–70% confluence were infected with 10^7^ PFU (plaque-forming units) of rAd5 per 15 cm plate. After 48 h, infected cells were collected, concentrated by low-speed centrifugation, resuspended in Tris HCl buffer (0.01 M Tris HCl, pH 8.0, 0.01 M NaCl, 5 mM EDTA) and disrupted by a triple freeze-thaw. The obtained suspension was centrifuged and purified by cesium chloride equilibrium density gradient centrifugation. The concentration of adenovirus was determined by a plaque-forming assay on HEK-293 cells.

### 2.4. Construction of Recombinant TC_0037

The gene encoding the full-length TC_0037 protein, along with six histidine tags (6x His), was cloned into the pET29b (Novagen, Madison, WI) expression vector at* Nde*I/*Xho*I restriction sites.* E. coli* BL21 (DE3), transformed with the designed plasmid, was grown in Luria-Bertani broth supplemented with ampicillin or kanamycin on a shaker at 37°C to an OD_600_ of 0.5. Protein expression from the pET29b-based plasmid was induced by 1 M isopropyl-*β*-D-thiogalactopyranoside (Roth, Karlsruhe, Germany) for 3 h at 25°C. The cleared lysate was subjected to chromatography on a nickel-equilibrated chelating Sepharose Fast Flow column according to the manufacturer's instructions (GE Healthcare, Rahway, NJ). Protein TC_0037 production was analyzed by western blotting with a monoclonal antibody targeting the histidine tag.

### 2.5. Vaccine Boost Preparation

The boosting squalene oil-in-water emulsion was prepared as described previously [40]. Briefly, a boosting vaccine formulation (200 *μ*L per dose) consisting of rTC_0037 protein (10 *μ*g/dose), immunostimulatory molecule MPLA (1 *μ*g/dose) (Sigma-Aldrich, St. Louis, MO), 50% squalene (Sigma-Aldrich, St. Louis, MO), 0.5% Tween 80, and 0.5% Span 85 in an isotonic phosphate buffer (Sigma-Aldrich, St. Louis, MO) was prepared by homogenization at 12,000 psi with a microfluidizer (Microfluidics, Newton, MA) and passed through a polysulfone filter (220 nm pore size; GE Healthcare, USA) for sterilization. The average diameter (108.3 +/− 8.7 nm) of the emulsion droplets was determined by Nanoparticle Tracking Analysis (NTA) using a NanoSight NS300 (Malvern Ltd., UK).

### 2.6. Chlamydia

*C. muridarum* (strain Nigg) ATCC VR-123 was grown in cycloheximide-treated McCoy cells (a hybrid cell line consisting of human synovial and mouse fibroblasts) as described previously [41, 42]. Chlamydial elementary bodies (EBs) were harvested, purified, quantified, and stored at −70°C in SPG buffer (sucrose/phosphate/glutamic acid: 0.2 M sucrose, 20 mM sodium phosphate, and 5 mM glutamic acid).

### 2.7. Ethics Statement

All animal work was undertaken in strict accordance with the recommendations of the National Standard of the Russian Federation (GOST R 53434-2009). The procedures used were approved by the Gamaleya Research Center of Epidemiology and Microbiology Institutional Animal Care. Female 6–8-week-old BALB/c mice obtained from the Animal Resource Center (Puschino breeding facility, Puschino, Moscow, Russia), accredited by the Association for Assessment and Accreditation of Laboratory Animal Care (AAALAC International), were maintained at the central animal facility of the Gamaleya Research Center of Epidemiology and Microbiology.

### 2.8. Immunization and Infection

**Figure 2:**
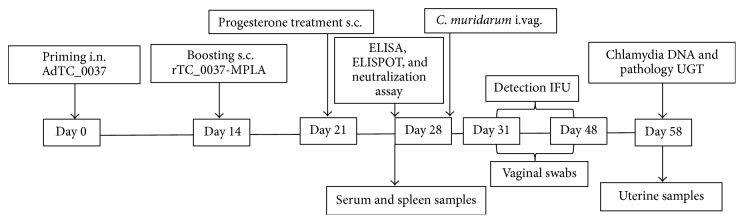
Study design for animal experiments.

Mice (*n* = 20 per group) were intranasally (i.n.) primed with 10^8^ PFU of adenoviral vector-expressed TC_0037 (AdTC_0037) in 100 *μ*L of sterile phosphate-buffered saline (PBS) or empty vector (Ad-null control). Prime-boost experimental groups of mice were further subcutaneously (s.c.) boosted with 200 *μ*L of rTC_0037-MPLA two weeks after priming (Figure 2). To evaluate vaccine protection, progesterone-treated mice (*n* = 10 per group) were intravaginally (i.vag.) infected with 10^6^ IFU (inclusion-forming units) of* C. muridarum* in 40 *μ*L of PBS as described previously [42].

### 2.9. Mouse Sampling

Blood (*n* = 5 per group) was collected from tails and centrifuged 2 weeks after boost immunization, and serum was harvested and stored at–20°C. To assess IFN-*γ* T cell responses, lymphocyte cultures from spleens (*n* = 5 per group) were prepared as described previously [40]. Briefly, spleens were removed aseptically from mice at day 14 after immunization. Spleen cells from mice infected with* C. muridarum* obtained at 14 days after infection were used as a positive control. Spleen cells (2 × 10^6^ per mL) were incubated with UV-killed* C. muridarum *or rTC_0037 for 24 h in complete RPMI-1640 (Gibco, Carlsbad, CA) containing 5% fetal bovine serum, 2 mM l-glutamine, and 1% penicillin-streptomycin (Gibco, Carlsbad, CA). Vaginal swabs were obtained at 3, 6, 9, 15, and 20 days after infection. Uterine samples for chlamydial DNA detection were collected in PBS and frozen at −70°C. Gross uterine pathology presenting as a hydrosalpinx was examined at day 30 after intravaginal* C. muridarum* challenge of immunized and naive mice.

### 2.10. Detection of TC_0037-Specific Antibodies in Serum

The antibody (IgG1 and IgG2a subclasses) response in serum was analyzed using an enzyme-linked immunosorbent assay (ELISA) with plates coated with rTC_0037 protein. Briefly, rTC_0037 protein was diluted to 10 *μ*g/mL in Na_2_CO_3_ (0.1 M, pH 9.5) and coated onto microtiter plates (NUNC, Rochester, NY). Nonspecific binding was blocked by using 0.1% bovine serum albumin (BSA) in PBS for 30 min at 37°C. Serum was diluted and titrated in 0.1% BSA in PBS and then incubated overnight at 4°C. Biotinylated anti-mouse IgG1 and IgG2a monoclonal antibodies (BioLegend, San Diego, CA), used at a dilution of 1 : 1000, were added to plates and incubated for 1 h at room temperature (20°C), followed by the addition of streptavidin-HPR and TMB substrate (all from BioLegend, San Diego, CA). The reaction was stopped 20 min later by adding 50 *μ*L/well of 1 M H_2_SO_4_. Absorbances at 450 nm were determined using a Multiskan EX microplate reader (Thermo Fisher Scientific Oy, Vantaa, Finland).

### 2.11. *In Vitro* Serum Neutralization Assay

A* C. muridarum* neutralization assay was performed in McCoy cell cultures [42]. Briefly, 100 *μ*L of* C. muridarum* EB suspension (1.4 × 10^5^ IFU/mL) in Dulbecco's modified Eagle's medium (DMEM, Paneco, Moscow, Russian) was added to 100 *μ*L of serial dilutions (from 1 : 16 to 1 : 256) of immune sera from vaccinated mice and incubated for 30 min at 37°C on a slowly rocking platform. Samples incubated with EB but without serum were used as an infection control. One hundred microliters of each dilution were then inoculated in duplicate into McCoy cells. DMEM containing 10% fetal bovine serum and 1 mg/mL of cycloheximide were added up to 1 mL to infected cells in 24-well plates with coverslips (12 mm in diameter). After centrifugation at 800 ×g for 1 h, infected cultures were incubated at 37°C for 48 h. After this, ethanol-fixed monolayers were stained with fluorescein isothiocyanate- (FITC-) conjugated monoclonal antibodies against chlamydial lipopolysaccharide (Nearmedic Plus, Moscow, Russia). Inclusion-containing cells were examined using a Nikon Eclipse 50i fluorescent microscope (Nikon, Amsterdam, Netherland) at 1350x magnification and counted to determine the percent infected cells in the monolayer.

### 2.12. Detection of Chlamydia and TC_0037-Specific IFN-*γ*-Producing T Cells

Chlamydia and TC_0037-specific IFN-*γ*-producing T cell enzyme-linked immunospot (ELISPOT) assays were performed on mouse splenocytes using a mouse IFN-gamma ELISPOT Ready-SET-Go! Kit (eBioscience, Inc., San Diego, CA) according to manufacturer's instructions. Briefly, IFN-*γ* capture antibody was coated onto ethanol-activated MultiScreen-IP Filter Plates (Millipore, Billerica, MA), incubated overnight at 4°C, and then washed with sterile PBS. CD4^+^ and CD8^+^ T cells were isolated from the spleens of vaccinated mice using CD4^+^ and CD8^+^ T cell separation columns (Cederlane, Burlington, Canada) according to manufacturer's protocols. The final concentration of enriched T cells was approximately 85%, as determined by flow cytometry; therefore, we hypothesized that there were enough antigen-presenting cells left to present antigens in the study. This assumption was confirmed in a separate set of experiments. Enriched cell suspensions were added to the coated plates at 2 × 10^5^ cells per well (in 100 *μ*L of culture medium) in the presence of UV-treated* C. muridarum* EB (positive control) at a final concentration of 10^4^ IFU/mL and rTC_0037 at a final concentration of 10 *μ*g/mL. The plates were incubated for 24 h before further washing. After this, plates were incubated with biotinylated anti-IFN-*γ* monoclonal antibodies. The spots were visualized with Avidin-HRP reagent and freshly prepared 3-amino-9-ethyl carbazole (AEC) substrate solution. Spots were calculated using an AID ELISpot Reader (Autoimmun Diagnostika GmbH, Strassberg, Germany).

### 2.13. Quantification of the Bacterial Loads in Chlamydia-Challenged Mice

Vaginal swabs were obtained at 3, 6, 9, 15, and 20 days after infection. The canal and exocervix were vigorously scraped, and swabbed material was transferred to DMEM and frozen immediately at −70°C. Bacterial loads were determined as previously described [41]. Thawed, undiluted samples (0.5 mL) were plated onto McCoy cells on coverslips (12 mm in diameter) in 24-well plates, and the infection was enhanced by centrifugation at 800 ×g for 1 h. After centrifugation, the inocula were removed, 1 mL of complete DMEM (DMEM with 10% fetal calf serum, 1% nonessential amino acids, 2 mM L-glutamine, 1% vitamins, and 0.5% glucose) was added, and the plates were incubated in a humidified incubator with 5% CO_2_ at 37°C for 48 h. Coverslips were incubated with FITC-conjugated monoclonal antibodies against chlamydial lipopolysaccharide (Nearmedic Plus, Moscow, Russia). Inclusion-containing cells were determined using a Nikon Eclipse 50i fluorescent microscope (Nikon, Amsterdam, Netherland) at 1350x magnification. The results were expressed as log_10_ IFU per vaginal swab.

### 2.14. Detection of Chlamydial DNA in Uteruses

Chlamydial DNA in uteruses was evaluated using a quantitative real-time PCR that allowed enumeration of the parasite's genome equivalents in infected tissue. Uterine samples were collected at day 30 after infection, homogenized in 1 mL of physiological solution, and frozen at −70°C. The prepared suspensions were lysed in 1 mL of lysis buffer (bioMérieux, Netherlands) and incubated with 40 *μ*L of proteinase K (Syntol, Moscow, Russia) at 60°C for 1 h. DNA was extracted with automated nucleic acid extractor NucliSENS easyMAG (bioMérieux, Zaltbommel, Netherlands). DNA contamination controls were included. The primers and TaqMan probe targeting the cryptic plasmid region were selected using Primer 3 (http://simgene.com/Primer3) and Oligo7 programs and are presented in Table 1.* C. muridarum* DNA was amplified using a Real-Time PCR Cycler CFX96 (Bio-Rad, Irvine, CA). The results are presented as percent positive mice in each group.

### 2.15. Pathology

The percent of mice with hydrosalpinges in each group was assessed at day 30 after* C. muridarum* challenge as described previously [43].

### 2.16. Statistical Analysis

The results were analyzed with the aid of GraphPad Prism 7.0 software (GraphPad software, San Diego, CA). Shapiro–Wilk test was used to determine normality of combined datasets from several similar experiments. The Mann–Whitney *U* test was used to evaluate differences in antibody titers in ELISA, specific IFN-*γ*-producing CD4^+^ and CD8^+^ T cells, and* Chlamydia* loads. An analysis of variance (ANOVA) test was used to compare neutralizing antibodies in experimental groups.

## 3. Results

### 3.1. Immunization with AdTC_0037 Results in the Production of TC_0037-Specific Antibodies of IgG2a and IgG1 Isotypes

**Figure 3:**
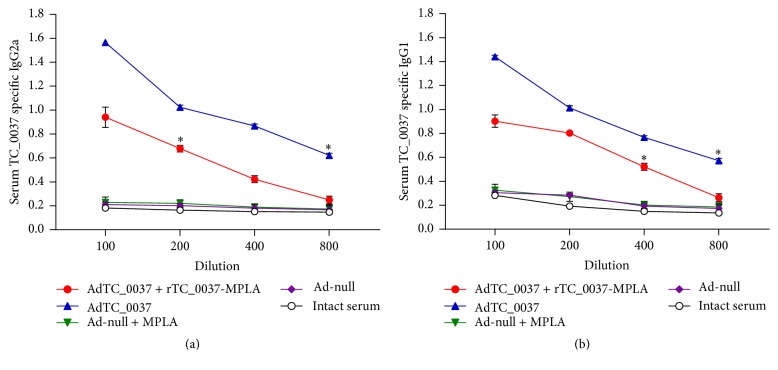
IgG2a and IgG1 antibody responses to TC_0037 following prime-boost immunization with AdTC_0037/rTC_0037-MPLA. BALB/c mice (*n* = 5 per group) were intranasally primed with Ad-TC_0037 followed by subcutaneous boosting with rTC_0037-MPLA. Sera were collected 2 weeks after the boost. TC_0037-specific IgG2a (a) and IgG1 (b) titers were determined by enzyme-linked immunosorbent assay and expressed as the mean ± SEM for groups of 5 mice from two experiments (^*∗*^*P* < 0.05).

The immunogenicity of the TC_0037 antigen was assessed in BALB/c mice immunized with TC_0037 expressed in an adenoviral vector (AdTC_0037), followed by subcutaneous immunization with recombinant TC_0037 and MPLA in a squalene nanoscale emulsion (rTC_0037-MPLA). Sera from mice immunized with an empty vector, with or without MPLA, and sera from intact mice were used as controls. AdTC_0037 immunization induced TC_0037-specific IgG2a and IgG1 antibodies with titers of 1 : 800 for both isotypes (*P* < 0.05) (Figure 3). Prime-boost immunization also resulted in an increase in specific IgG2a and IgG1 antibodies, but the titers were lower than those in the AdTC_0037-alone group (1 : 400 and 1 : 800, resp.). Thus, TC_0037 expressed in the adenovirus vector induced specific antibody responses in mice.

### 3.2. Prime-Boost Immunization with AdTC_0037/r TC_0037-MPLA Induces Antibodies That Neutralize* C. muridarum* Infectivity* In Vitro*

**Figure 4:**
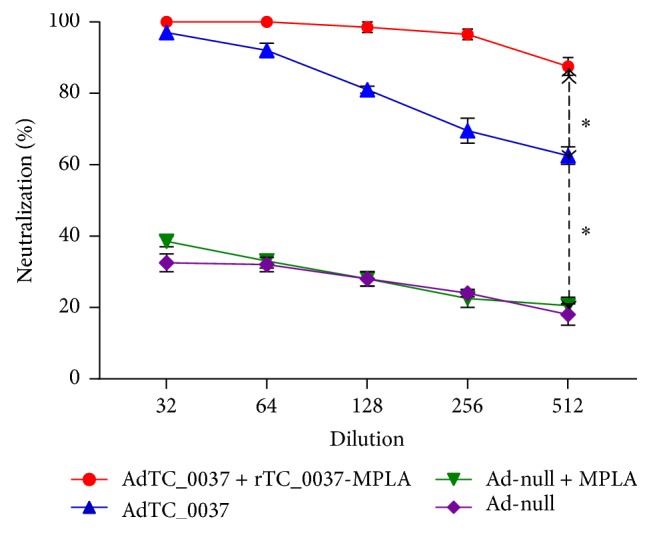
Antibodies against* Chlamydia muridarum* TC_0037 neutralize chlamydia infectivity* in vitro*.* C. muridarum* elementary bodies were opsonized with sera from immunized or control mice (*n* = 5) at different dilutions and used to infect McCoy cell monolayers. Inclusions were counted 48 h after infection. Neutralization activity was determined by measuring the reduction in the number of inclusions generated by antibody-opsonized elementary bodies. The graphs present the percent neutralization as an average of experiments performed in triplicate with standard deviations (^*∗*^*P* < 0.01).

In the experiments described above, we demonstrated that immunization with AdTC_0037/rTC_0037-MPLA or AdTC_0037 alone induced TC_0037-specific antibodies of IgG2a and IgG1 isotypes. Next, we assessed the ability of serum antibodies to neutralize pathogen infectivity* in vitro*.* C. muridarum* EBs were preincubated with serum from immunized mice and then used to infect McCoy cell monolayers. Chlamydial inclusions were counted under the microscope 48 h after incubation. The results are presented in Figure 4. We found that sera from prime-boost immunized mice effectively neutralized EB infectivity* in vitro* up to 90% at a dilution of 1 : 512. In the group immunized with AdTC_0037, the reduction in infectivity reached 65% (dilution 1 : 512). Overall, our results suggest that immunization with AdTC_0037 induces antibodies with high potential to neutralize* C. muridarum* infection* in vitro*. Interestingly, the neutralizing potential of specific antibodies from mice immunized with AdTC_0037/rTC_0037-MPLA was higher than that of antibodies in mice immunized with AdTC_0037 alone (*P* ≤ 0.01). This might contribute to higher protection with the prime-boost approach.

### 3.3. Induction of Specific IFN-*γ*-Producing CD4^+^ and CD8^+^ T Cells in Mice Immunized with AdTC_0037/rTC_0037-MPLA and Infected with* C. muridarum*

**Figure 5:**
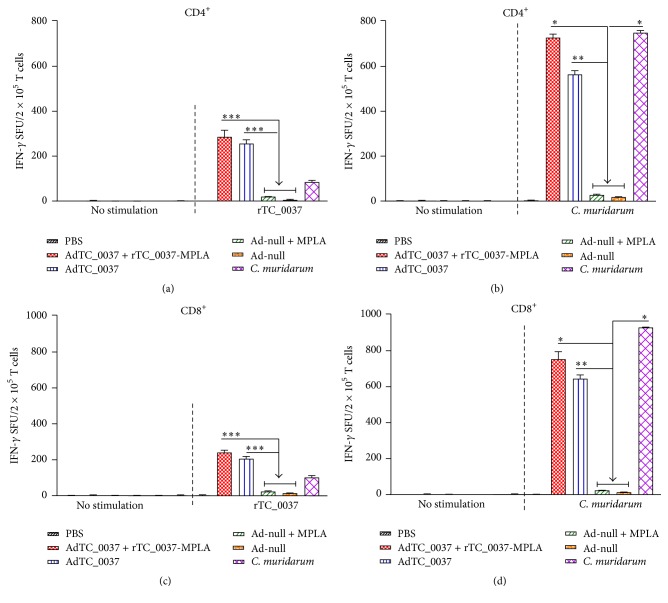
Prime-boost immunization with AdTC_0037/rTC0037-MPLA induces specific IFN-*γ*-producing CD4^+^ and CD8^+^ responses. BALB/c mice (*n* = 5 per group) were immunized intranasally with Ad-TC_0037 and boosted subcutaneously with rTC_0037-MPLA. Mice infected intravaginally with* Chlamydia muridarum* were used as controls. Two weeks after the boost, CD4^+^ and CD8^+^ T cells were isolated and stimulated* in vitro* with TC_0037 or UV-killed* C. muridarum*. ELISPOT counts (spot-forming units [SFUs]) in response to TC_0037 and* C. muridarum *were analyzed. Results are presented as the mean SFU per 2 × 10^5^ splenocytes ± SEM (^*∗*^*P* ≤ 0.005; ^*∗∗*^*P* ≤ 0.01; ^*∗∗∗*^*P* ≤ 0.05 relative to Ad-null-MPLA immunized mice).

Since IFN-*γ* has been found to play a major role in mediating control of* Chlamydia* infection, we measured IFN-*γ* responses to rTC_0037 and UV-killed* C. muridarum* in immunized and infected mice to evaluate the potential of TC_0037 to prime T cell responses to* Chlamydia*. TC_0037 and* C. muridarum*-specific IFN-*γ*^+^ CD4^+^ and CD8^+^ T cell responses were assessed by ELISPOT 2 weeks after final immunization or infection. As shown in Figure 5, rTC_0037 and UV-killed* C. muridarum* stimulation triggered robust IFN-*γ* production in CD4^+^ and CD8^+^ T cells derived from mice immunized with AdTC_0037/rTC_0037-MPLA or AdTC_0037 alone, but not with the control adenovirus (Ad-null+MPLA, Ad-null). UV-killed* C. muridarum* stimulation induced IFN-*γ* CD4^+^ and CD8^+^ production in immunized mice at the same level as that in* C. muridarum*-infected mice (Figures 5(b) and 5(d)). However, CD4^+^ and CD8^+^ T cell responses to TC_0037 were higher in the immunized groups (Figures 5(a) and 5(c)). Our results indicate that immunization with rTC_0037 resulted in the induction of robust TC_0037-specific IFN-*γ*+ CD4^+^ T cell responses that were comparable to those induced in naturally infected mice (Figure 5(a)). Additionally, it potentiates the induction of higher levels of IFN-*γ*+ TC_0037-specific CD8^+^ T cells compared to those in* C. muridarum*-infected mice (Figure 5(c)).

### 3.4. Immunization with AdTC_0037 and AdTC_0037/rTC_0037-MPLA Enhances the Clearance of* C. muridarum* in the Vagina

**Figure 6:**
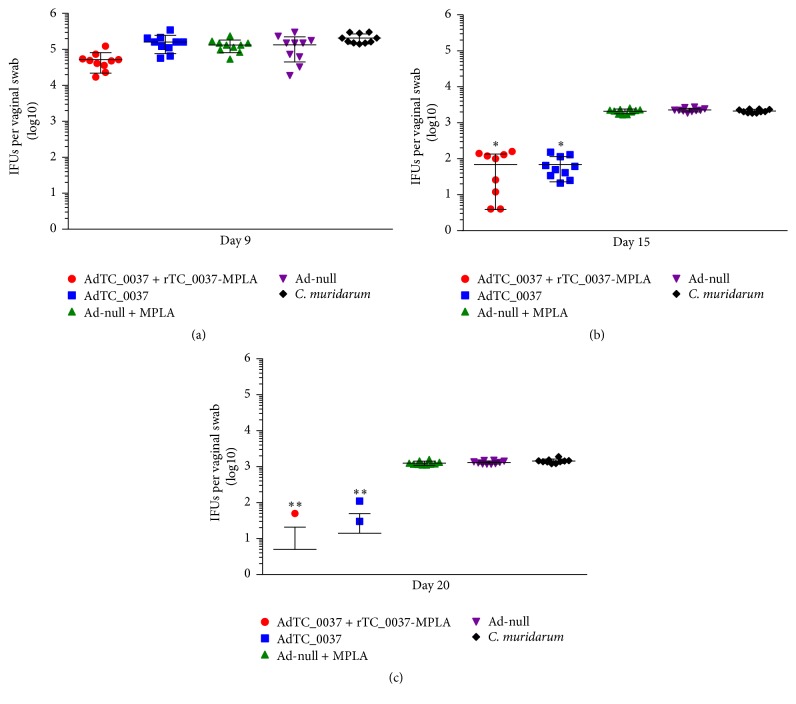
Prime-boost immunization with Ad-TC_0037/rTC0037-MPLA enhances clearance of intravaginal* Chlamydia muridarum *in BALB/c mice. Depo-Provera-treated BALB/c mice (*n* = 10 per group) were infected intravaginally with 10^6^ IFU of* C. muridarum* strain Nigg 2 weeks after the boost. The vaginal vaults of mice were sampled using individual swabs at days 3 to 20 after challenge, and vaginal chlamydial loads were quantified using an* in vitro* infection assay and immunofluorescent microscopy. Individual and median values of* C. muridarum* loads measured at days 9 (a), 15 (b), and 20 (c) after infection are presented (^*∗*^*P* ≤ 0.005, ^*∗∗*^*P* ≤ 0.01).

BALB/c mice were i.vag. infected with* C. muridarum *at day 14 after final immunization, and cervicovaginal swabs were obtained from mice (Figure 2) on days 3, 7, 9, 15, and 20 following challenge. To quantify resistance to infection, recoverable IFU were compared between the groups (Figure 6). There were no statistically significant differences between the groups at days 3 and 7 (data not shown) and at day 9 (Figure 6(a)) after challenge. However, prime-boost immunization with AdTC_0037/rTC_0037-MPLA significantly reduced chlamydial IFUs at day 15 (Figure 6(b), ^*∗*^*P* < 0.005). Almost complete eradication of infection (9 of 10 mice cleared infection in AdTC_0037/rTC_0037-MPLA group) was observed at day 20 after infection (Figure 6(c), ^*∗∗*^*P* < 0.01), whereas eradication following immunization with AdTC_0037 and in the infection control was not observed. These results show that prime-boost immunization with AdTC_0037/rTC_0037-MPLA reduced the magnitude and duration of* C. muridarum* vaginal shedding after infection.

### 3.5. Immunization with AdTC_0037/rTC_0037-MPLA Reduces Chlamydia Loads in the Upper Genital Tract

**Figure 7:**
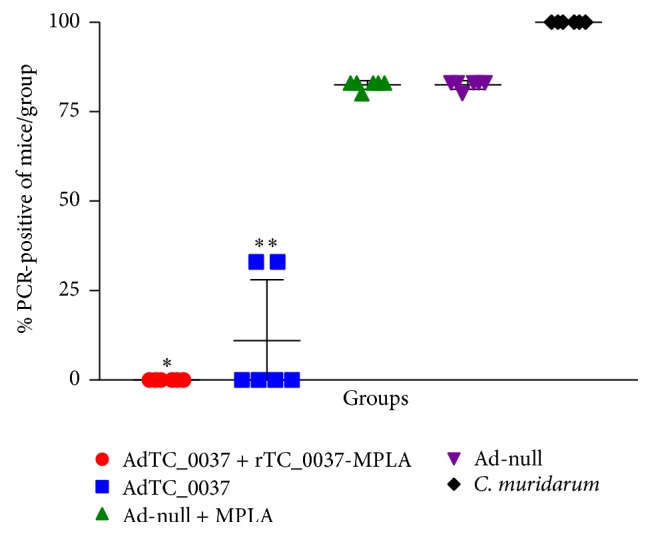
Prime-boost immunization with AdTC_0037/rTC_0037-MPLA reduces chlamydia loads in the upper genital tracts of BALB/c mice. Uteruses were collected at day 30 after infection, and* Chlamydia muridarum* loads were determined by polymerase chain reaction. A 100% reduction in bacterial infection was observed in the prime-boost vaccinated group compared to the control groups (^*∗*^*P* < 0.01; ^*∗∗*^*P* < 0.001).

To assess the effects of vaccination on control of ascending infection, we analyzed the levels of chlamydial DNA in uterine samples at day 30 after infection. Immunization of mice with AdTC_0037 in both groups resulted in a decrease in* C. muridarum *DNA in the upper genital tract of mice, and this effect was more pronounced in the mice immunized with AdTC_0037/rTC_0037-MPLA (Figure 7). Therefore, we confirmed that prime-boost immunization with TC_0037 antigen successfully inhibited upper genital* C. muridarum *infection* in vivo*.

### 3.6. Immunization with AdTC_0037 Results in Decreased Pathology in the Upper Genital Tract

**Figure 8:**
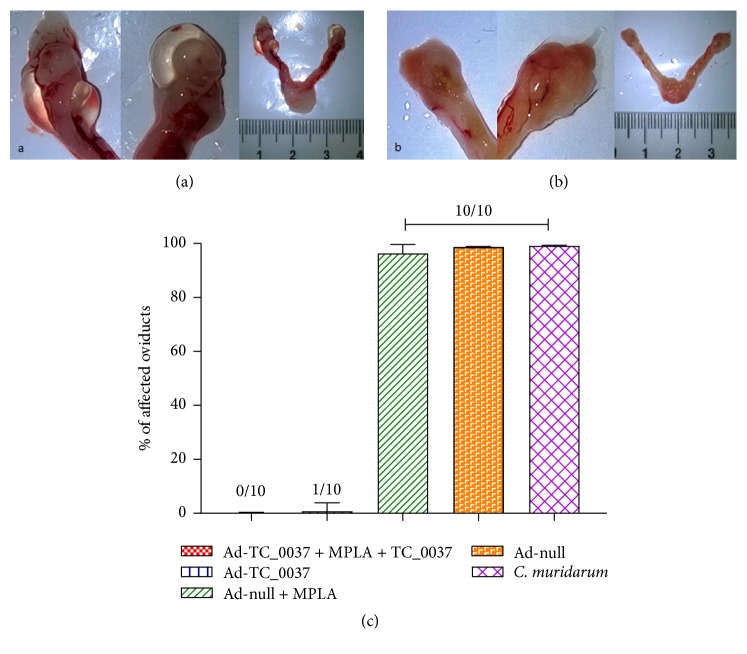
Prime-boost immunization with AdTC_0037/rTC_0037-MPLA affects oviduct pathology in* Chlamydia muridarum* BALB/c mice. Mice were immunized with AdTC_0037/rTC_0037-MPLA, challenged intravaginally with* C. muridarum*, and sacrificed 30 days after infection. Oviducts were analyzed for the presence of a hydrosalpinx. (a) Hydrosalpinges in ovaries collected from unvaccinated mice, compared to the complete absence of oviduct pathology in vaccinated mice (b). (c) Percent of mice with reduced pathology in AdTC_0037-immunized groups.

One of the primary goals for an effective chlamydial vaccine is to reduce or prevent upper genital tract pathology following lower tract infection. To assess the ability of TC_0037 immunization to reduce* Chlamydia*-induced immunopathology of the ovarian ducts, BALB/c mice were immunized as shown in Figure 2 and challenged i.vag. with 10^6^ IFU of* C. muridarum*. Animals were sacrificed at day 30 after infection to assess the development of hydrosalpinx. As shown in Figure 8, mice vaccinated with AdTC_0037 or AdTC_0037/rTC_0037-MPLA did not develop upper genital tract pathology, whereas mice in the control groups did (Figure 8(c)). Thus, immunization with AdTC_0037 resulted in complete abrogation of pathology in the upper genital organs of BALB/c mice infected i.vag. with* C. muridarum*.

## 4. Discussion

Despite decades of research and considerable progress made in recent years, an efficacious* C. trachomatis* vaccine remains elusive [44]. As previous successful uses of an adenovirus-vectored vaccine against* C. muridarum* (expressing CPAF and MOMP) have been reported [45–48], in this study we developed a novel adenovirus-based vaccine expressing T3SS* Chlamydia* antigen TC_0037 and evaluated its protective potential in a* C. muridarum* mouse model. To enhance protection by our vaccine candidate, we utilized a heterologous prime-boost immunization regimen, using a combination of intranasal priming with adenovirus-expressing TC_0037 and subcutaneous boost with recombinant TC_0037 and MPLA mixed in squalene. Prime-boost immunization of mice with adenovirus-expressed TC_0037 and rTC_0037/MPLA elicited serum antibodies against TC_0037 of isotypes IgG2a and IgG1 (Figure 3). In addition, serum from immunized mice neutralized chlamydial infection* in vitro* (Figure 4). Mice vaccinated with TC_0037 and challenged with* C. muridarum* showed a reduction in both bacterial shedding and* Chlamydia*-induced fallopian tube pathology.

TC_0037 protein forms a conserved and abundant TTSS needle structure and may be a strong candidate protein for inclusion in a pan-serovar* Chlamydia* vaccine [12]. We show here for the first time that TC_0037, a T3SS needle protein from* C. muridarum, *represents a good vaccine candidate, as it is able to induce specific antibody and T cell responses and decrease bacterial loads and pathology of the upper genital tract.

To date, few studies have examined the use of T3SS proteins as antigens to vaccinate against* Chlamydia*. Recently, intranasal immunization with a mixture of three T3SS components, CopB, CopD, and CT584, with CpG resulted in the production of specific neutralizing antibodies and decreased chlamydial infection and* Chlamydia-*induced pathology [18]. In contrast, T3SS components have been used intensively as vaccine candidates against other Gram-negative bacteria with considerable success. Significant protection against* in vivo* challenge with* Shigella* has been demonstrated after vaccination with a* Shigella* CopB ortholog in combination with other T3SS antigens [49]. Antibodies to the T3SS tip proteins LcrV in* Yersinia *spp. and PcrV in* Pseudomonas aeruginosa* have been shown to block these infections [47, 48]. In our study, we provided evidence that immunization with another component of the chlamydial T3SS stimulated strong humoral and cellular immune responses and afforded protection against intravaginal* Chlamydia* challenge.

Cell-mediated immune responses have been documented as critical for protection and clearance of* Chlamydia* infection. A large amount of data has been accumulated on the role of CD4^+^ T cells in protection against genital infection in previous* Chlamydia* vaccine studies [50, 51]. The importance of IFN-*γ* to* Chlamydia* control* in vivo* has also been demonstrated [52, 53]. In our study, immunization with AdTC_0037 and rTC_0037-MPLA induced robust, specific IFN-*γ* CD4^+^ and CD8^+^ T cell responses (Figure 5). In these studies, we used CD4^+^ and CD8^+^ enriched T cell populations with purity of 85%. It should be noted that the remaining 15% of non-T cells could also contain cells that produce IFN-gamma. Besides, differences in the remaining subpopulations of antigen-presenting cells in different groups of mice could also affect the resulting IFN-gamma production. Future studies employing more rigorous protocols of CD4^+^ and CD8^+^ T cells isolation will help to elucidate the role of CD4^+^ and CD8^+^ T cells in the response to TC_0037. Prime-boost immunization also coinduced significant levels of TC_0037-specific IgG2a and IgG1 antibodies that had higher neutralizing activity than those in response to AdTC_0037 immunization, and they afforded better protection.

As reported in other studies [54, 55], enhanced clearance of* Chlamydia* is dependent upon CD4^+^ T cell responses. However, antibodies play a contributing role in the resolution of primary infections and protect from reinfection [56–58]. CD4^+^ and CD8^+^ T cells have been reported to act cooperatively with antibodies [59–61]. For example, passive transfer of immune serum protects mice against* Chlamydia* genital infection only in the presence of CD4^+^ T cells [55]. Several studies have linked antibodies to protection against upper genital pathology [60–63]. Antibodies of IgG2a isotype mediate effector functions, including antibody-dependent cell-mediated cytotoxicity (ADCC), with evidence suggesting that this effector function facilitates early clearance of chlamydial infections. Furthermore, ADCC is associated with enhanced antigen presentation, with the potential to amplify CD4^+^ T cell responses [64]. In our study, we did not elucidate the direct contribution of specific IgG2a antibodies to better protection from* Chlamydia* challenge, but we observed that a higher level of neutralizing antibody activity in AdTC_0037/rTC_0037-MPLA-immunized mice was associated with better protection. This is in agreement with previous findings in mice regarding the role of neutralizing antibodies in protection against* Chlamydia *[63]. Better neutralizing activity in AdTC_0037/rTC_0037-MPLA-immunized mice is possibly connected with the boosting component of our vaccine; however, further studies on the role of rTC_0037-MPLA in neutralization of chlamydial infectivity and protection will be helpful. Previously, antibodies raised against T3SS translocators, CopB and CopD, were reported to inhibit chlamydial infection* in vitro*, suggesting that antibodies directed at these proteins block an essential component of T3SS virulence [18]. Serum collected from mice immunized with TC_0037 reduced pathogen infectivity, suggesting that these antibodies were directed against surface-exposed epitopes on TC_0037. Although the mechanism of neutralization remains to be elucidated, it was presumably because antibodies rendered the T3SS inactive, preventing host cell infection.

An important characteristic of a* Chlamydia* vaccine is its ability to decrease bacterial shedding for reduced transmission and to produce an immune response that prevents* Chlamydia*-induced immunopathology. During the course of a chlamydial infection in mice, bacterial shedding occurs for approximately 14–35 days before being cleared from the lower genital tract [18]. Based on mouse models of infection, pathology in the upper genital tract is believed to occur as a result of an ascending infection from the lower genital tract. To assess the ability of TC_0037 immunization to reduce bacterial shedding, vaginal swabs were collected and bacterial loads were quantified. Prime-boost immunization with AdTC_0037/r TC_0037-MPLA significantly reduced chlamydial IFUs per swab at day 15 and caused almost complete eradication (9 of 10 mice) of infection at day 20 after infection (Figure 6). It also decreased bacterial loads in the upper genital tract (Figure 7). Both results indicate the high potential of our vaccine candidate to provide protection against intravaginal chlamydial infection.

Previously our group has successfully used Ad5 for design of vaccines against influenza [10],* Bacillus anthracis* [65], Ebola virus [30, 31], and some other pathogens. Ad5 vectors are commonly used among the adenoviruses due to a wide range of their well-described attractive features. They are replication-defective and can be easily transfected and accumulated in cell culture [66]. They allow incorporation of large antigens that can be secreted by host cell. They can be additionally modified by the components boosting natural immunity. It was also shown that they are effective even after single immunization [65]. Overall, Ad5 is a very promising delivery platform actively exploited worldwide. In this study, we show that Ad5 can provide an effective delivery system for* Chlamydia* vaccine candidate TC_0037. However, we did not compare the efficacy of our delivery system to other possible variants such as virus-like particles. This question requires additional studies in future.

It should be noted that we used adenovirus as a delivery system for our antigen only once in our protocol avoiding possible complications due to preexisting adenoviral immunity. However, taking into account the fact that our adenoviral delivery system is intended as a new platform for a range of novel vaccines to different pathogens, the possibility of preexisting immunity should be taken into consideration. In our study, we employed Ad5 genome modification with mannose-binding lectin to improve the efficacy of our vaccine and to escape limitations connected with preexisting adenoviral immunity. However, the practical efficacy of this approach expects confirmation in preclinical and clinical trials. The safety of adenoviral vaccines is another question that should be addressed, especially for widespread diseases like* Chlamydia* infection. Recently, our group published the results of successful clinical trial of Ad5-based Ebola vaccine candidate that confirmed safety of this vaccine [30, 31]. Adenovirus-based influenza vaccine has successfully passed phase I clinical trials in the USA and proved to be safe for humans and highly immunogenic for the influenza A H5N2 virus [67]. Besides, there are a great number of successful safety reports published recently by different groups. Overall, safety of Ad-based vaccines can be accepted as reasonably established at present.

Because the development of oviduct pathology and complications are major concerns in individuals infected with* C. trachomatis*, we examined upper urogenital tracts following pathogen challenge to determine whether vaccination could prevent* Chlamydia*-induced pathology. Indeed, it completely eradicated pathology in the upper genital tract in mice vaccinated with AdTC_0037/rTC_0037-MPLA or AdTC_0037 alone (Figure 8).

Overall, evaluation of AdTC_0037/rTC_0037-MPLA vaccination in* C. muridarum* infection model in mice demonstrates a potentially effective vaccine candidate, which provides B and T cell immune responses and protects from genital tract infection and pathology. However, to proceed closer to a clinical trial, there is a need for testing of our vaccine candidate in a model employing* C. trachomatis.* Besides, nonrodent animal models with better resemblance to* Chlamydia* infection in humans can afford higher level of immunological and physiological relevance. Finally, long-term protection studies are also highly urgent to complete the profile of protection for our vaccine candidate.

## 5. Conclusion

The resolution of genital* C. muridarum* infection coupled with protection against* Chlamydia*-induced pathology suggests that prime-boost immunization with AdTC_0037/rTC_0037-MPLA affords a significant degree of protection and TC_0037 can be considered for further evaluation as a vaccine candidate. We are currently investigating the potential of our vaccine candidate to protect against intravaginal* C. trachomatis* infection in mice in our recently developed murine model [42].

## Figures and Tables

**Table 1:** Primers for the detection of *Chlamydia muridarum*.

Name	Gene	5′-3′ sequence
C.mur-F	P MoPn	Forward 5′-TGCGATAGAAACAATTCCCTGAGT -3′
C.mur-R		Reverse 5′-TGCTTTAGAAAAGATTGGGCTATTG-3′
C.mur_up_FAM		Probe FAM-AGCTGCACGAACTTGTTTGGTGCCTTCT- RTQ1
